# Targeted Genome Editing of Sweet Orange Using Cas9/sgRNA

**DOI:** 10.1371/journal.pone.0093806

**Published:** 2014-04-07

**Authors:** Hongge Jia, Nian Wang

**Affiliations:** Citrus Research and Education Center, Department of Microbiology and Cell Science, University of Florida, Lake Alfred, Florida, United States of America; National Institute of Plant Genome Research, India

## Abstract

Genetic modification, including plant breeding, has been widely used to improve crop yield and quality, as well as to increase disease resistance. Targeted genome engineering is expected to contribute significantly to future varietal improvement, and genome editing technologies using zinc finger nucleases (ZFNs), transcription activator-like effector nucleases (TALENs), and clustered regularly interspaced short palindromic repeat (CRISPR)/Cas9/single guide RNA (sgRNA) have already been successfully used to genetically modify plants. However, to date, there has been no reported use of any of the current genome editing approaches in sweet orange, an important fruit crop. In this study, we first developed a novel tool, Xcc-facilitated agroinfiltration, for enhancing transient protein expression in sweet orange leaves. We then successfully employed Xcc-facilitated agroinfiltration to deliver Cas9, along with a synthetic sgRNA targeting the *CsPDS* gene, into sweet orange. DNA sequencing confirmed that the *CsPDS* gene was mutated at the target site in treated sweet orange leaves. The mutation rate using the Cas9/sgRNA system was approximately 3.2 to 3.9%. Off-target mutagenesis was not detected for *CsPDS*-related DNA sequences in our study. This is the first report of targeted genome modification in citrus using the Cas9/sgRNA system—a system that holds significant promise for the study of citrus gene function and for targeted genetic modification.

## Introduction

Genetic modification, including plant breeding, has been widely used to improve crop yield, quality and disease resistance. In particular, targeted genome engineering promises to contribute greatly to future varietal improvement. Genome editing technologies that use zinc finger nucleases (ZFNs), transcription activator-like effector nucleases (TALENs), and clustered regularly interspaced short palindromic repeat (CRISPR)/Cas9/single guide RNA (sgRNA) (hereafter named Cas9/sgRNA) have been successfully used to genetically modify plants. Through the fusion of the FokI nuclease domain to a set of customized zinc finger proteins, ZFNs can be used to cleave a target DNA site, which is subsequently subjected to error-prone nonhomologous end joining (NHEJ) that leads to targeted mutagenesis. ZFNs have thus far been used successfully for genome modification in soybean [1], *Arabidopsis thaliana* [2]–[6], *Zea mays* [7], and tobacco [8]. More recently, TALEN technology has emerged as a more attractive approach for genome engineering, owing to its more tractable nature [9], [10]. TALENs can be used to target any desirable site based on a simple code that results from the repeat-variable di-residue (RVD) sequences found within a conserved TALE repeat, with each RVD specifically binding to a corresponding nucleotide [11], [12]. To date, TALENs have been harnessed for genome editing in *A. thaliana* [13], [14], rice [15], *Nicotiana tabacum* [16], barley [17], and *Brassica oleracea* L. var. capitata L [18].

Most recently, the Cas9/sgRNA system has been undergoing rapid development and is now regarded as another very promising method for genome engineering [9]. In nature, the CRISPR/Cas system serves as the adaptive immune system of prokaryotes. The CRISPR locus contains a characteristic array of repeat sequences interspersed by spacer sequences derived from foreign genetic elements emanating from previous virus or plasmid DNA invasions. In some cases, DNA from a newly invading virus or plasmid DNA will match the DNA in the CRISPR “spacer,” and a CRISPR/Cas9/crRNA-mediated response is mounted and the foreign DNA is destroyed [19]. The type II CRISPR/Cas system from *Streptococcus pyogenes* SF370 has been developed for targeted genome engineering [20]–[22]. This system is composed of the Cas9, Cas1, Cas2, and Csn1 proteins, as well as CRISPR RNA (crRNA) and trans-activating CRISPR RNA (tracrRNA). In response to foreign nucleic acid assaults, tracrRNA is transcribed and hybridizes with pre-crRNA to form a functional crRNA with the aid of the Cas9 protein. The mature crRNA:tracrRNA duplex guides Cas9 to the targeted protospacer region, which is upstream of a protospacer-adjacent motif (PAM). The crRNA spacer hybridizes with the protospacer to facilitate Cas9-mediated cleavage within the protospacer. The CRISPR/Cas system has been simplified from a three-component system to a two-component, Cas9/single guide RNA (sgRNA) system, in which the Cas9 protein binds to a synthetic sgRNA that contains a fusion of the crRNA and tracrRNA elements.

The Cas9/sgRNA system is gaining in popularity due to its simplicity and affordability [23]. Whereas both ZFNs and TALENs demand elaborate designs and the assembly of individual DNA-binding proteins for each DNA target [1], [5], [9], [15], [24], the Cas9/sgRNA system requires changes only in the recombinant sgRNA for target specificity rather than in the Cas9 protein [20], [25]–[28]. Cas9/sgRNA technology has been successfully used for genome editing in rice, wheat, Arabidopsis, tobacco and sorghum [25]–[28].

However, none of the aforementioned genome editing approaches have been reported for use in citrus. Citrus is the most economically important and extensively grown fruit tree crop in the world, with sweet orange accounting for approximately 60% of citrus production in 2009 [29]. Despite its importance, the genetic improvement of citrus is limited by the slow growth and maturation of this crop, as well as by crop pollen incompatibility, polyembryony, and parthenocarpy [30]. Thus, it is important to develop new methods for targeted citrus genome modification (especially for sweet orange). In this study, we show that a customizable sgRNA can be used to direct the Cas9-mediated, sequence-specific genetic modification of sweet orange using Xcc-facilitated agroinfiltration.

## Materials and Methods

### Plant materials

Three-year-old sweet orange (*Citrus sinensis*, cultivar Valencia) plants were cultivated in a quarantine greenhouse facility at the Citrus Research and Education Center, Lake Alfred, FL, U.S.A, at temperatures ranging from 25 to 30°C. A specific permit was issued by the Division of Plant Industry, Florida Department of Agriculture and Consumer Service, for the location and activities. Before agroinfiltration, the plants were pruned to produce uniform shoots.

### Plasmid construction

DNA sequences for all primers in this study are provided in Table S1 in File S1. The 35S promoter of CaMV, derived from the binary vector pBI121 (accession number AF485783), was cloned into pCambia1380 (accession number AF234301) using *Hin*dIII and *Bam*HI to produce 1380-35S. pBI121 was obtained from The Arabidopsis Information Resource (Stock#: CD3-388). Cas9 containing a Flag tag at its N-terminus and a nuclear localization signal at its C-terminus was amplified from Addgene plasmid 42230 using a pair of primers, Cas9-5-*Bam*HI and Cas9-3-*Eco*RI [20]. After *Bam*HI-*Eco*RI digestion, the PCR product was inserted downstream of the CaMV 35S promoter to produce the construct 1380-35S-Cas9. A Nos terminator was amplified using the primers NosT-5-*Eco*RI and NosT-3-*Xho*I-*Asc*I. The PCR products were digested with *Eco*RI and *Asc*I and placed downstream of the Cas9 coding region to obtain 1380-Cas9. The sgRNA scaffold portion was amplified from Addgene plasmid 41819, using the primers sgRNA-5-*Bam*HI and sgRNA-3-*Sac*I [22], and inserted into pBI121 following digestion with *Bam*HI and *Sac*I to form 121-sgRNA.

The CaMV 35S promoter fragment was amplified using the primers CaMV35-5-*Xho*I and sgRNA-CsPDS2, and the sgRNA-NosT fragment was amplified using the primers sgRNA-CsPDS1 and NosT-3-*Asc*I. Using three-way ligation, *Xho*I-digested CaMV35S and *Asc*I-cut sgRNA-NosT were cloned into *Xho*I-*Asc*I-treated 1380-Cas9 to construct 1380-Cas9:sgRNA targeting the *CsPDS* gene.

### Xcc-facilitated agroinfiltration of sweet orange leaves

**Figure 1:**
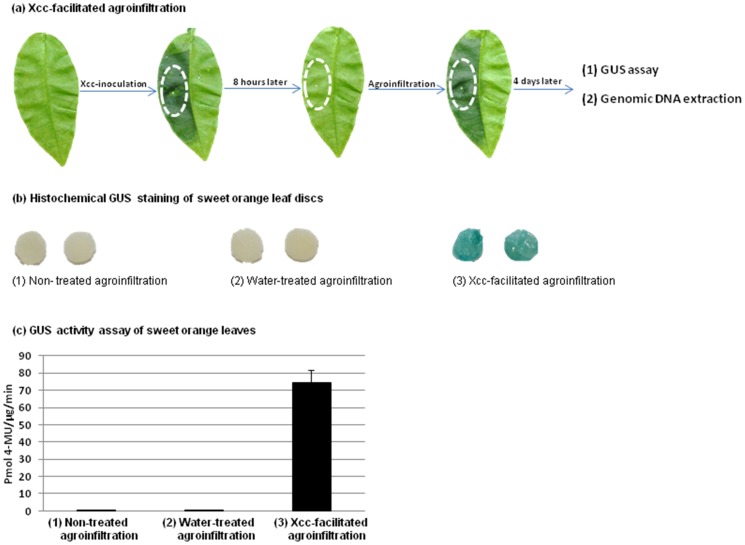
Xcc-facilitated agroinfiltration of sweet orange leaf. (a) Scheme of the Xcc-facilitated agroinfiltration method. The sweet orange leaf area, circled by a white oval, was first inoculated with an Xcc re-suspension and then treated 8 hours later with agroinfiltration. Four days later, the oval-circled leaf tissue was analyzed. (b) GUS staining to show that Xcc-facilitated agroinfiltration increased GUS expression in sweet orange leaves. Sweet orange leaves were infiltrated with *Agrobacterium tumefaciens* harboring pCambia1301, which contains a GUS construct. Eight hours before agroinfiltration, leaves were left non-treated (1), treated with tap water (2), or with Xcc (5×10^8^ CFU/ml) re-suspended in sterile tap water (3). Four days after agroinfiltration, GUS staining was carried out to assay GUS expression. (c) Quantitative GUS assay performed to confirm that Xcc-facilitated agroinfiltration enhanced GUS expression in sweet orange leaves. pCambia1301-transformed *Agrobacterium* was infiltrated into sweet orange leaves. Eight hours before agroinfiltration, leaves were left non-treated (1), treated with tap water (2), or with Xcc (5×10^8^ CFU/ml) re-suspended in sterile tap water (3). Four days after agroinfiltration, GUS activity was quantified. The experiment was repeated three times. The error bars indicate standard deviations (SD).

Sweet orange leaves were inoculated with either tap water or a culture of actively growing *Xanthomonas citri* subsp. citri (Xcc) re-suspended in sterile tap water (5×10^8^ CFU/ml). Eight hours later, the same inoculated leaf areas were subjected to agroinfiltration as described previously [31], with modifications (Fig. 1a). Recombinant *Agrobacterium tumefaciens* cells were cultured in 3 ml Luria broth (LB) medium with appropriate antibiotics at 28°C. One hundred microliters of overnight culture was grown in 100 ml fresh LB medium with 10 mM 2-(N-morpholino) ethanesulfonic acid (MES), pH5.6, and 40 μM acetosyringone (AS), as well as the appropriate antibiotics. Upon reaching OD_600_ = 0.8, the inoculum was harvested and resuspended in MMA solution (10 mM MgCl_2_, 10 mM MES, pH 5.6 and 200 μM AS) to a final OD_600_ of 1.0. The suspension was left at room temperature for 2 h and infiltrated into the same area previously inoculated by Xcc. As a control, citrus leaves were subjected to agroinfiltration in the absence of prior Xcc inoculation.

### Histochemical GUS staining and quantitative GUS assay

**Figure 2:**
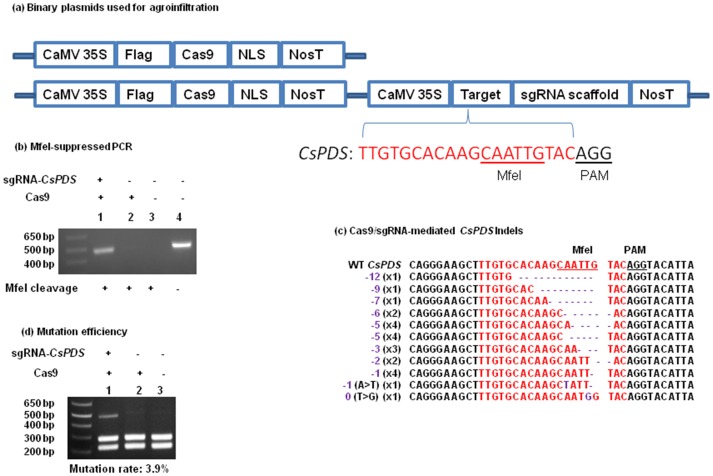
Targeted genome engineering in sweet orange using the Cas9/sgRNA system. (a) Scheme of the binary vectors 1380-Cas9 and 1380-Cas9:sgRNA. A Flag tag and a nuclear localization signal (NLS) were fused to the Cas9 N-terminus and C-terminus, respectively. Cas9 catalyzes the cleavage of the sgRNA-targeting sequence immediately upstream of the PAM. Here, Cas9/sgRNA was employed to target the *CsPDS* gene (red). The *Mfe*I restriction site and the protospacer adjacent motif (PAM) are underlined. (b) Selective PCR amplification of mutagenized *CsPDS* genes was used to detect the Cas9/sgRNA-induced mutation *in planta*. PCR amplification was conducted using the primers CsPDS-5-P1 and CsPDS-3-P2, which flank the target site within the *CsPDS* gene (Table S1 in File S1). Lanes 1-3, the template genomic DNA was digested with *Mfe*I. Lane 4, nondigested genomic DNA was used as a template. The PCR product in lane 1 resulted from Cas9/sgRNA-induced disruption of the *Mfe*I site and was therefore cloned into the PCR-BluntII-TOPO vector (Life Technologies) for sequencing. (c) Targeted mutations induced by Cas9/sgRNA in the *CsPDS* gene in sweet orange. Sequences of mutant variants of the *CsPDS* gene obtained from the clones constructed using the PCR product from lane 1 in Fig. 2b were aligned with the wild type sequence (top). The sgRNA-targeted *CsPDS* sequence is shown in red, and the mutations are shown in purple. (d) Measurement of the mutation rate of the *CsPDS* gene induced by Cas9/sgRNA. Genomic DNA was extracted from three samples (co-expression of Cas9 and sgRNA; expression of Cas9 alone; no expression of Cas9 and sgRNA), and subjected to PCR amplification using the primers CsPDS-5-P1 and CsPDS-3-P2. The PCR products were digested with *Mfe*I and analyzed by DNA gel electrophoresis (Lane 1, co-expression of Cas9 and sgRNA; Lane 2, expression of Cas9 alone; Lane 3, no expression of Cas9 and sgRNA). The mutation rate was calculated by dividing the intensity of the uncut band by the intensity of all the bands in each lane.

Xcc-facilitated agroinfiltration was carried out with sweet orange leaves using *A. tumefaciens* cells transformed with Cas9/sgRNA binary plasmids (Fig. 2a) or with pCambia1301 (accession number AF234297), which carries a GUS construct [31]. Four days after agroinfiltration with pCambia1301-transformed *Agrobacterium*, GUS staining was performed as described previously (Fig. 1a) [32]. The β-Glucuronidase Reporter Gene Staining Kit (Sigma-Aldrich) was used for GUS staining following the manufacturer's protocols. After overnight staining, the citrus leaf samples were destained and subsequently photographed.

The GUS activity assay was performed as described previously [33], with modifications. Thirty milligrams of leaf tissue was ground in 300 μl of GUS extraction buffer (50 mM NaPO_4_ (pH 7.0), 1 mM Na_2_EDTA, 0.1% SDS, 0.1% Triton X-100, 10 mM DTT) using a mortar and pestle. The ground material was centrifuged at 4°C for 15 min at top speed (12,000× *g*) in a tabletop centrifuge. After centrifugation, 200 μl of supernatant was collected, and the amount of protein was measured using Bradford reagent (Bio-Rad, Hercules, CA). Twenty-five microliters of extract was mixed with 225 μl of GUS extraction buffer containing 1 mM 4-methyl-umbelliferyl glucuronide (MUG), and then the mixture was incubated at 37°C. After 30 min and 90 min, 100 μl of the reaction was added to 900 μl of 0.2 M Na_2_CO_3_, and the fluorometric values were determined using a VersaFluor Fluorometer (BioRad). The GUS activity is expressed as pmol of 4-methyl-umbelliferone (MU) per μg protein per min.

### Genomic DNA extraction

Four days after Xcc-facilitated agroinfiltration with 1380-Cas9:sgRNA-transformed *Agrobacterium*, genomic DNA was extracted from the sweet orange leaves using a Wizard Genomic DNA Purification Kit (Fig. 1a) (Promega) according to the manufacturer's protocol. The genomic DNA was dissolved in 100 μl of distilled deionized water, and the concentration was determined using a spectrophotometer.

### Selective PCR amplification of the mutagenized *CsPDS* gene

To confirm the targeted modification of the *CsPDS* gene (accession number XM_006492918), 400 ng of genomic DNA was digested with *Mfe*I for 3 hours at 37°C. Using the digested genomic DNA as the template, PCR was performed with the Phusion DNA polymerase (New England Biolabs) and a pair of primers, CsPDS-5-P1 and CsPDS-3-P2, flanking the target site in *CsPDS*. The PCR products were ligated into the PCR-BluntII-TOPO vector (Life Technologies), and 28 random colonies were selected for DNA sequencing.

### Measurement of mutation rates

The method used to measure the mutation rate was previously reported by Shan et al. [28]. After PCR amplification using non-digested genomic DNA as a template, the PCR products were treated with *Mfe*I and analyzed on an agarose gel. By using AlphaImager EP (AlphaInnotech) software, the intensity of the ethidium bromide-stained DNA bands was quantified after background subtraction, and the band intensities were summed to obtain the total intensities. To measure the mutation frequency, the intensity of the uncut band was divided by the total intensity.

### Analysis of potential off-target sequences

Potential off-target sequences were identified by searching the sweet orange genome database via the BLASTN tool [29] against the sgRNA target site within the *CsPDS* gene (TTGTGCACAAGCAATTGTAC). A total of 46 off-target sequences were found, of which 23 contained an *Mfe*I site within the aligned sequence (Table S2 in File S1). Fourteen regions out of the 23 that lacked an *Mfe*I site close to the target site were chosen for analysis. Attempts were made to amplify these regions by PCR using the primers listed in Table S1 in File S1. The eight regions amplified by PCR were analyzed by agarose gel electrophoresis, either with or without prior digestion by *Mfe*I.

## Results and Discussion

### Xcc-facilitated agroinfiltration enhances GUS expression in citrus leaves

Previously, it has been difficult to implement agroinfiltration-mediated transient expression in citrus leaves (Fig. 1) [34]. Importantly, our results indicated that GUS expression was dramatically enhanced in sweet orange leaves using Xcc-facilitated agroinfiltration (Fig. 1b, Fig. 1c) (i.e., initial inoculation with Xcc followed eight hours later by agroinfiltration with recombinant *A. tumefaciens* cells containing a GUS gene (Fig. 1a)). In contrast, there was very low GUS activity in sweet orange leaves when agroinfiltration was performed alone, or when leaves were pretreated with tap water (Fig. 1b, Fig. 1c). Therefore, Xcc pre-treatment is very important for enhanced transient protein expression in citrus leaves.

One possible mechanism to explain the fact that Xcc pre-treatment could significantly increase the transient expression of GUS in agroinfiltrated sweet orange leaves is that in Xcc-inoculated citrus leaves, the PthA4 effector is known to be translocated from Xcc into plant cell nuclei, where it activates downstream target genes. This leads to excessive cell division (hyperplasia) and cell enlargement (hypertrophy) [35]–[37]. Interestingly, citrus epicotyl segments are being widely used as explants for *Agrobacterium*-mediated citrus transformation, owing to the higher proportion of actively dividing cells, which are presumed to be more susceptible to T-DNA, in epicotyl tissue [34]. It seems possible that in the case of Xcc-facilitated agroinfiltration, Xcc is eliciting excessive cell division, which might mimic the fast dividing epicotyl segment. Such rapidly dividing cells may also be more susceptible to *A. tumefaciens* transformation, thereby resulting in the enhanced GUS expression observed in treated sweet orange leaves (Fig. 1b, Fig. 1c).

### Targeted sweet orange genome modification mediated by Cas9/sgRNA via Xcc-facilitated agroinfiltration

To test the potential of the Cas9/sgRNA system to induce genetic modification in citrus, Xcc-facilitated agroinfiltration was employed to deliver the Cas9 endonuclease and an sgRNA. We first constructed the binary vector p1380-Cas9:sgRNA (Fig. 2a, Fig. S1 in File S1), which contains both Cas9 and an sgRNA targeting the *CsPDS* gene. *CsPDS* encodes a phytoene desaturase with homologs in rice, Arabidopsis and *Nicotiana benthamiana*, which have been successfully genetically modified using Cas9/sgRNA [25], [28], [38]. The 35S promoter of CaMV was used to drive the expression of Cas9 and the sgRNA targeting *CsPDS*. Cas9 was fused with a nuclear localization signal at the C-terminus (Fig. 2a) [20].

We expressed Cas9 and the *CsPDS*-targeting sgRNA in sweet orange leaf tissue using Xcc- facilitated agroinfiltration. Four days after agroinfiltration, sweet orange genomic DNA was extracted from the treated leaf areas (Fig. 1a) and digested using the *Mfe*I restriction enzyme. The target sequence within the *CsPDS* gene contains an *Mfe*I restriction site, which will be mutated if Cas9/sgRNA works as predicted. Thus, selective amplification of mutagenized *CsPDS* genes was used to verify targeted genome engineering in sweet orange. As expected, the *CsPDS* PCR product (480 bp) was observed in control leaves not treated with *Mfe*I (Fig. 2b, lane 4). However, after *Mfe*I digestion, the 480-bp band was only detected in the *Mfe*I-cleaved genomic DNA of sweet orange leaves co-expressing Cas9 and the *CsPDS*-targeting sgRNA (Fig. 2b, lane 1) and not in sweet orange leaves expressing Cas9 alone or control leaves (Fig. 2b, lane 2 and lane 3). Two additional replicates showed similar results (Fig. S2 in File S1). This result indicated that Cas9/sgRNA successfully induced mutations in the targeted *CsPDS* gene.

The PCR products were purified from lane1 (co-expression of Cas9 and *CsPDS*-targeting sgRNA) and cloned for sequencing (Fig. 2c, Fig. S3 in File S1). A total of twenty-eight colonies were selected for sequencing. The sequencing results showed twenty-four clones with indels in the targeted sequence, which abolished the *Mfe*I restriction site within the target region (Fig. 2c, Fig. S3 in File S1). Further detailed analysis revealed that the indels could be grouped into eleven different types, including deletions (from 1 bp to 12 bp) and nucleotide substitutions (Fig. 2c, Fig. S3 in File S1). Although the *CsPDS* gene was clearly targeted by Cas9/sgRNA (Fig. 2c, Fig. S3 in File S1), there were no visible albino spots on the treated sweet orange leaves. This result is consistent with results from *Arabidopsis* and *Nicotiana benthamiana*, whose leaves also did not show visible albino spots after Cas9/sgRNA-mediated *PDS* targeting via agroinfiltration [38].

To calculate the efficiency of *CsPDS* mutation caused by Cas9/sgRNA in citrus, PCR products were amplified from the genomic DNA of Cas9/sgRNA-treated and control leaf tissues, digested with *Mfe*I, and subjected to gel electrophoresis. Based on the ratio of the intensities of uncut DNA bands to the total intensity of both cut and uncut bands from three independent experiments [28], the mutation rate was estimated to be 3.2%, 3.4% and 3.9%(Fig. 2d, Fig. S4 in File S1). In a previous report [38], the mutation rate caused by Cas9/sgRNA was 5.6% in *Arabidopsis* protoplasts and 37.7% in *N. benthamiana* protoplasts, whereas the mutation frequency was 2.7% in agroinfiltrated *Arabidopsis* leaves and 4.8% in *N. benthamiana* leaves. Thus, the mutation rate in sweet orange leaves is comparable to that induced by Cas9/sgRNA after leaf agroinfiltration in *Arabidopsis* and *N. benthamiana*.

It should be noted that the 35S promoter of CaMV was employed to drive sgRNA expression (Fig. 2a, Fig. S1 in File S1). It has been previously reported that the 35S promoter of CaMV could be utilized to transcribe the sequence of interest with no extraneous sequence at the 5′ end if the sequence was inserted at the transcription start site of the 35S promoter of CaMV [39]. Using the same scheme, the sgRNA was inserted at the transcription start site of the CaMV 35S promoter (Fig. S1 in File S1). In comparison to the plant U6 promoter, which constrains the first nucleotide at the transcription start site to be ‘G’ [28], the 35S promoter of CaMV seems to allow more flexibility in the sgRNA design, owing to a lack of constraint with regard to the first nucleotide. This was confirmed by the results.

We also analyzed potential off-target mutagenesis caused by Cas9/sgRNA. We identified a total of 46 potential off-target sequences by searching the *C. sinensis* genome database using the 20-bp target sequence of the *CsPDS* gene in BLASTN (Table S2 in File S1). Those off-target sequences contain 13 to 17 nucleotides identical to the *CsPDS*-targeting sequence (Table S2 in File S1). Fourteen regions out of the 46 identified sequences contained *Mfe*I sites and did not have additional *Mfe*I sites in close proximity to the off-target sequences. Thus, we selected those 14 sequences for *Mfe*I-suppressed PCR analysis. Only 8 sequences were amplified successfully using PCR (Fig. S5 in File S1). None of the 8 amplicons showed evidence of Cas9/sgRNA-induced *Mfe*I restriction site loss similar to that observed with the *CsPDS* target sequence (Fig. 2, Fig. S5 in File S1). Thus, off-target mutagenesis was not detected for *CsPDS* in our study, even though we could not rule out potential off-target mutagenesis in other sequences. This is most likely a case-by-case situation that depends on whether high-identity off-target sequences are present in non-targeted genes and whether we could select unique target sequences during our bioinformatic analysis, considering the recent findings of off-target mutagenesis induced by Cas9/sgRNA [40].

Our data complements recent studies in which the Cas9/sgRNA system was successfully developed to modify the genomes of plants such as Arabidopsis, tobacco, rice, wheat, and sorghum [41]. For the first time, the sweet orange genome has proven to be readily targeted via the Cas9/sgRNA system, with the aid of Xcc-facilitated agroinfiltration. It must be kept in mind that this study did not produce stable Cas9/sgRNA-transformed sweet orange. Thus, it is worth confirming that Cas9/sgRNA can also be employed to modify the sweet orange genome in transgenic plants. Given that Cas9/sgRNA works in transgenic citrus, Cas9/sgRNA-mediated citrus genome editing holds significant promise for studies of citrus gene function and for targeted genetic modification. In conclusion, Cas9/sgRNA appears to be a potentially valuable tool for creating new citrus cultivars with beneficial traits for both growers and consumers.

## Supporting Information

File S1 **The Supporting Information contains Tables S1–S2 and Figures S1–S5. Figure S1, The sequence of the CaMV 35S promoter-CsPDS-targeting sgRNA-NosT of the Cas9/sgRNA construct.** The CaMV 35S promoter is shown in blue. The guide sequence is shown in red. The sgRNA scaffold is shown in purple. The Nos terminator is shown in green. The transcription start site is marked by an arrow. **Figure S2, Two replicates of the experiment presented in** **Figure 2 (b)** **.** Restriction-enzyme-digestion-suppressed PCR was used to detect the Cas9/sgRNA-induced mutation *in planta*. PCR amplification was conducted using the primers CsPDS-5-P1 and CsPDS-3-P2, which flank the target site within the *CsPDS* gene. Lanes 1-3, the template genomic DNA was digested with *Mfe*I. Lane 4, nondigested genomic DNA was used as a template. The PCR product in lane 1 resulted from Cas9/sgRNA-induced disruption of *Mfe*I, which indicates the expected disruption of the *Mfe*I site within the *CsPDS* gene. M, 1 kb DNA ladder. **Figure S3, Two replicates of the experiment presented in** **Figure 2(d)** **.** Measurement of the mutation rate of the *CsPDS* gene induced by Cas9/sgRNA. After PCR amplification of the targeted PDS region, the products were subjected to *Mfe*I digestion. After separation on an agarose gel, the intensities of the bands were quantified using AlphaImager EP. The mutation rate was calculated by dividing the intensity of the uncut band by the intensity of all the bands in the lane. M, 1 kb DNA ladder. **Figure S4, The representative indel chromatograms of the** ***CsPDS12*** **mutations inducted by Cas9/sgRNA.** The target sequence within the *CsPDS* gene is highlighted by an orange rectangle. **Figure S5, Analysis of potential off-target sequences of the** ***CsPDS*** **-targeting Cas9/sgRNA by** ***Mfe*** **I-suppressed PCR.** (a) Eight potential off-target sequences were amplified by PCR when non-digested genomic DNA was used as the template. (b) When *Mfe*I-digested genomic DNA was used, no PCR products or alleviated PCR products were observed. (c) After selective PCR amplification of mutagenized *CsPDS* genes, only the PCR product from the *CsPDS* gene showed resistance to *Mfe*I digestion. These results indicated that the *Mfe*I restriction sites in the 8 potential off-target sequences were not disrupted by Cas9/sgRNA cleavage and NHEJ repair. **Table S1, Primers used for Cas9/sgRNA-mediated genome engineering in sweet orange. Table S2, Forty-six potential off-target sequences of the** ***CsPDS*** **gene in the sweet orange genome.**
(PDF)
