# Supplementary material for: Targeted Genome Editing of Sweet Orange Using Cas9/sgRNA
Source: PLoS One. 2014 Apr 7;9(4):e93806. doi: 10.1371/journal.pone.0093806 (PMC3977896; doi:10.1371/journal.pone.0093806)
Supplement: File S1 — The Supporting Information contains Tables S1–S2 and Figures S1–S5. Figure S1, The sequence of the CaMV 35S promoter-CsPDS-targeting sgRNA-NosT of the Cas9/sgRNA construct. The CaMV 35S promoter is shown in blue. The guide sequence is shown in red. The sgRNA scaffold is shown in purple. The Nos terminator is shown in green. The transcription start site is marked by an arrow. Figure S2, Two replicates of the experiment presented in Figure 2 (b) . Restriction-enzyme-digestion-suppressed PCR was used to detect the Cas9/sgRNA-induced mutation in planta. PCR amplification was conducted using the primers CsPDS-5-P1 and CsPDS-3-P2, which flank the target site within the CsPDS gene. Lanes 1-3, the template genomic DNA was digested with MfeI. Lane 4, nondigested genomic DNA was used as a template. The PCR product in lane 1 resulted from Cas9/sgRNA-induced disruption of MfeI, which indicates the expected disruption of the MfeI site within the CsPDS gene. M, 1 kb DNA ladder. Figure S3, Two replicates of the experiment presented in Figure 2(d) . Measurement of the mutation rate of the CsPDS gene induced by Cas9/sgRNA. After PCR amplification of the targeted PDS region, the products were subjected to MfeI digestion. After separation on an agarose gel, the intensities of the bands were quantified using AlphaImager EP. The mutation rate was calculated by dividing the intensity of the uncut band by the intensity of all the bands in the lane. M, 1 kb DNA ladder. Figure S4, The representative indel chromatograms of the CsPDS12 mutations inducted by Cas9/sgRNA. The target sequence within the CsPDS gene is highlighted by an orange rectangle. Figure S5, Analysis of potential off-target sequences of the CsPDS -targeting Cas9/sgRNA by Mfe I-suppressed PCR. (a) Eight potential off-target sequences were amplified by PCR when non-digested genomic DNA was used as the template. (b) When MfeI-digested genomic DNA was used, no PCR products or alleviated PCR products were observed. (c) After sele [file pone.0093806.s001.pdf]

Supplementary Table 1. Primers used for Cas9/sgRNA-mediated genome engineering in sweet orange

Primer name	Primer sequence from 5' to 3'	Note
Cas9-5-BamHI	AGGTGGATCCGGTTGGACCGGTGCCACCATG	
Cas9-3-EcoRI	TGATCAGCGAGCTCTAGGAATTCTTA	
NosT-5-EcoRI	AGGATCCACCGGTGCACGAATTCCGAATTTCCCCGATCGTTCAA	
NosT-3-XhoI-AscI	AGGCGCGCCATTTAAATCTCGAGCCGATCTAGTAACATAGATGA	
sgRNA-5-BamHI	TCGACTGGATCCGGTACCAAG	
sgRNA-3-SacI	AGGTGAGCTCTTCGCCCTTTAATGCCAACTT	
CaMV35S-5-XhoI	ACTCGAGACTAGTACCATGGTGGACTCCTCTTAA	
sgRNA-CsPDS2	TTGTGCACAACCTCTCCAAATGAAATGAACTTC	5'-phosphorylated
sgRNA-CsPDS1	GCAATTGTACGTTTTAGAGCTAGAAATAGCAA	5'-phosphorylated
NosT-3-AscI	ACCTGGGCCCGGCGCGCCGATCTAGTAACATAGATGA	
CsPDS-5-P1	AGGATTCTTCATTTTCAATGA	
CsPDS-3-P2	TGAACATTTCAATTACTTAGG	
7290-5-P1	TCCACTGGAACTGGTGGAATCACT	
7290-3-P2	TGATCATGGATATAAGAAGACT	
2370-5-P1	TACCCACAATCACAAGGATAAAGT	
2370-3-P2	TGACTAAGGATTGAGCCAGGTTCG	
4890-5-P1	AGACATCATCCAAGGGTACTCT	
4890-3-P2	TGACTGAACTTCCTACTTTCATG	
3808-5-P1	ACGATCTATTGGCAACATGCCAAA	
3808-3-P2	AGCTGGTGTAGGTGTCCTTTGTTC	
3020-5-P1	TGGGATCGATTATTGTTGGATTCA	
3020-3-P2	ACACCAAAACCCTTGTTAACAAATC	
5862-5-P1	TCATCCAACCGGATTGATATGCTC	
5862-3-P2	ACAAACACGGTAGCAAATGCTCCC	
9370-5-P1	ACCTTCTATCCCATCTTATCCTCAC	
9370-3-P1	ACACAACATACACAGGAGCAAGCA	
5100-5-P1	TGTGGTGCTTGTCCTAATTAGCAC	
5100-3-P2	ATACTCCCTGATTGCTGCAAGTCA	
6180-5-P1	TGTCCAAATTGAGCCCGAATTAAG	
6180-3-P2	TCATCTAATCTCACCTACCTCTTC	
2600-5-P1	AGTGAATGTCGTTTCATTTCACTG	
2600-3-P2	TCATACATTTCACATGACCCACTC	
8390-5-P1	TGAGCTTATCACAGAGCCCAATTC	
8390-3-P2	TCCATGTATGAAGACTCCACCCAA	
5080-5-P1	ACTGGCTGAATGATGGGCAAACCT	
5080-3-P2	TGCTCGTGTTCTATGTCGCTACAA	
0160-5-P1	TGTTGAAACATCTAGCAACCTGTAG	
0160-3-P2	ACGTATCGTACAATAATGGGTTGA	
2870-5-P1	TGCTTGTGCTTCACCTTCGTGTGA	
2870-3-P2	TGCTTCTCCGTGACCACACAACGC	

Supplementary Table 2. Forty-six potential off-target sequences of the *CsPDS* gene in the sweet orange genome

Off-target site	Off-target sequence	Test by MfeI-suppressed PCR
Cs9g07290	GAGAGCACAAGCAATTGTAC	Yes
Cs9g12370	CATGACACAAGCAATTGTAG	Yes
Cs7g14890	TGACACACAAGCAATTGTAA	Yes
Cs5g10470	CCATGCACAAGCAATTGCAC	No
Cs5g05850	CTGTGCACAAGCAATCGTGC	No
orange1.1t03808	GATGACACAAGCAATTGTTT	Yes
orange1.1t03020	TTTTCAACAAGCAATTGTAT	Yes
orange1.1t05862	CACTTCGCAAGCAATTGTAC	Yes
orange1.1t01105	ATCTGCACAAGCAATTTACA	No
orange1.1t00848	TTGTGCACAAGCACTTTGGA	No
orange1.1t00315	TCGAGCACAAGCAATTGCCA	No
Cs9g09370	AACCCTGCAAGCAATTGTAC	Yes
Cs9g05100	GAAGACCCAAGCAATTGTAC	Yes
Cs8g17760	AAATGCACAAGCAATTAACA	No
Cs8g16180	TAACATCCAAGCAATTGTAC	Yes
Cs8g12600	TGTGGCACAAGCAATTGAAT	Yes
Cs7g18390	AAGGATCCAAGCAATTGTAC	Yes
Cs7g09470	TTTTGCACAAGCAATTACAT	No
Cs7g04320	TTTTCCACAAGCAATTGTGT	No
Cs7g01860	TAGTGCACAAGCAATATGAC	No
Cs6g15500	ATGTGCACAAGCAAACCAAG	No
Cs6g05090	TAGCACACAAGCAATTGTCA	No
Cs6g02660	CCCTGCGCAAGCAATTGTAC	No
Cs5g31970	ACTTGCACAAGCAATTTACA	No
Cs5g20160	TTGTGCACAAGCAGTTTCTT	No
Cs5g18400	CTGTGCACAAGCAAACACTA	No
Cs5g11660	ACAATCACAAGCAATTGTTC	No
Cs5g10520	TGGTGCACAAGCAATCACCA	No
Cs4g12540	TTGTGCACAAGCACCCATTG	No
Cs4g12530	TTGTGCACAAGCACCCATTG	No
Cs3g25770	AGGTGCACAAGCAATATCAA	No
Cs3g25080	AACTTCACAAGCAATTGTGA	Yes
Cs3g21130	TGAATCACAAGCAATTGTGC	No
Cs3g20160	CAGTAGACAAGCAATTGTAA	Yes
Cs3g19160	GATTGCACAAGCAATTAGCA	No
Cs3g12870	TCTACCACAAGCAATTGTGA	Yes

Supplementary Table 2. Forty-six potential off-target sequences of the *CsPDS* gene in the sweet orange genome (continued)

Off-target site	Off-target sequence	Test by MfeI-suppressed PCR
Cs3g07250	TTGTGCACAAGCATTCCAAT	No
Cs2g29170	TTGTGCACAAGCATCTTAAA	No
Cs2g11120	TTGTGCACAAGCATAAACCA	No
Cs2g06350	TTATGCACAAGCAATTATAG	No
Cs2g06110	TCATGCACAAGCAATTAAGA	No
Cs2g04990	AAATATACAAGCAATTGTAA	No
Cs2g04980	AAATATACAAGCAATTGTAA	No
Cs1g18550	AGCAGCACAAGCAATTGAGT	No
Cs1g16510	TAGTGCACAAGCAATACGAC	No

The *Mfe*I site is shown in green; Mismatching bases are shown in red.

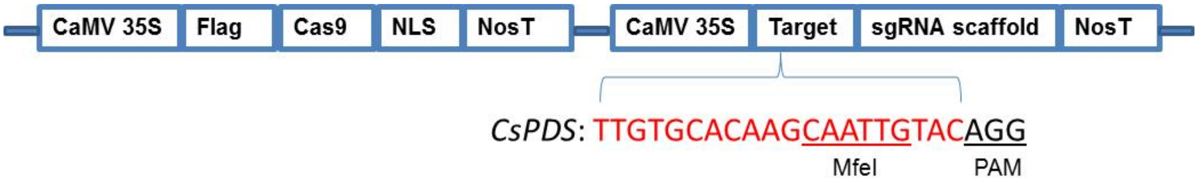

ctcgagACTAGTACCATGGTGGACTCCTCTTAAAGCTTGCATGCCTGCAGGTCCCCAGATTAGCCTTTTCAATTTCAGAAAG
AATGCTAACCCACAGATGGTTAGAGAGGCTTACGCAGCAGGTCTCATCAAGACGATCTACCCGAGCAATAATCTCCAGG
AAATCAAATACCTTCCCAAGAAGGTTAAAGATGCAGTCAAAAGATTCAGGACTAACTGCATCAAGAACACAGAGAAAGA
TATATTTCTCAAGATCAGAAGTACTATTCCAGTATGGACGATTCAAGGCTTGCTTCACAAACCAAGGCAAGTAATAGAGA
TTGGAGTCTCTAAAAAGGTAGTTCCCACTGAATCAAAGGCCATGGAGTCAAAGATTCAAATAGAGGACCTAACAGAACT
CGCCGTAAAGACTGGCGAACAGTTCATACAGAGTCTCTTACGACTCAATGACAAGAAGAAAATCTTCGTCAACATGGTG
GAGCACGACACACTTGTCTACTCCAAAAATATCAAAGATACAGTCTCAGAAGACCAAAGGGCAATTGAGACTTTTCAACA
AAGGGTAATATCCGGAAACCTCCTCGGATTCCATTGCCCAGCTATCTGTCACTTTATTGTGAAGATAGTGGAAAAGGAAG
GTGGCTCCTACAAATGCCATCATTGCGATAAAGGAAAGGCCATCGTTGAAGATGCCTCTGCCGACAGTGGTCCCAAAGA
TGGACCCCCACCCACGAGGAGCATCGTGGAAAAAGAAGACGTTCCAACCACGTCTTCAAAGCAAGTGGATTGATGTGAT
ATCTCCACTGACGTAAGGGATGACGCACAATCCCACTATCCTTCGCAAGACCCTTCCTCTATATAAGGAAGTTCATTTCAT
TTGGAGAGGTTGTGCACAAGCAATTGTACGTTTTAGAGCTAGAAATAGCAAGTTAAAATAAGGCTAGTCCGTTATCAAC
TTGAAAAAGTGGCACCGAGTCGGTGCTTTTTTTCTAGACCCAGCTTTCTTGTACAAAGTTGGCATTAAAGGGCGAAGAGC
TCGAATTTCCCCGATCGTTCAAACATTTGGCAATAAAGTTTCTTAAGATTGAATCCTGTTGCCGGTCTTGCGATGATTATC
ATATAATTTCTGTTGAATTACGTTAAGCATGTAATAATTAACATGTAATGCATGACGTTATTTATGAGATGGGTTTTTATG
ATTAGAGTCCCGCAATTATACATTTAATACGCGATAGAAAACAAAATATAGCGCGCAAACTAGGATAAATTATCGCGCGC
GGTGTCATCTATGTTACTAGATCggcgcgcc

Supplementary Figure S1. The sequence of the CaMV 35S promoter-CsPDS-targeting sgRNA – NosT of the Cas9/sgRNA construct. The CaMV 35S promoter is shown in blue. The guide sequence is shown in red. The sgRNA scaffold is shown in purple. The Nos terminator is shown in green. The transcription start site is marked by an arrow.

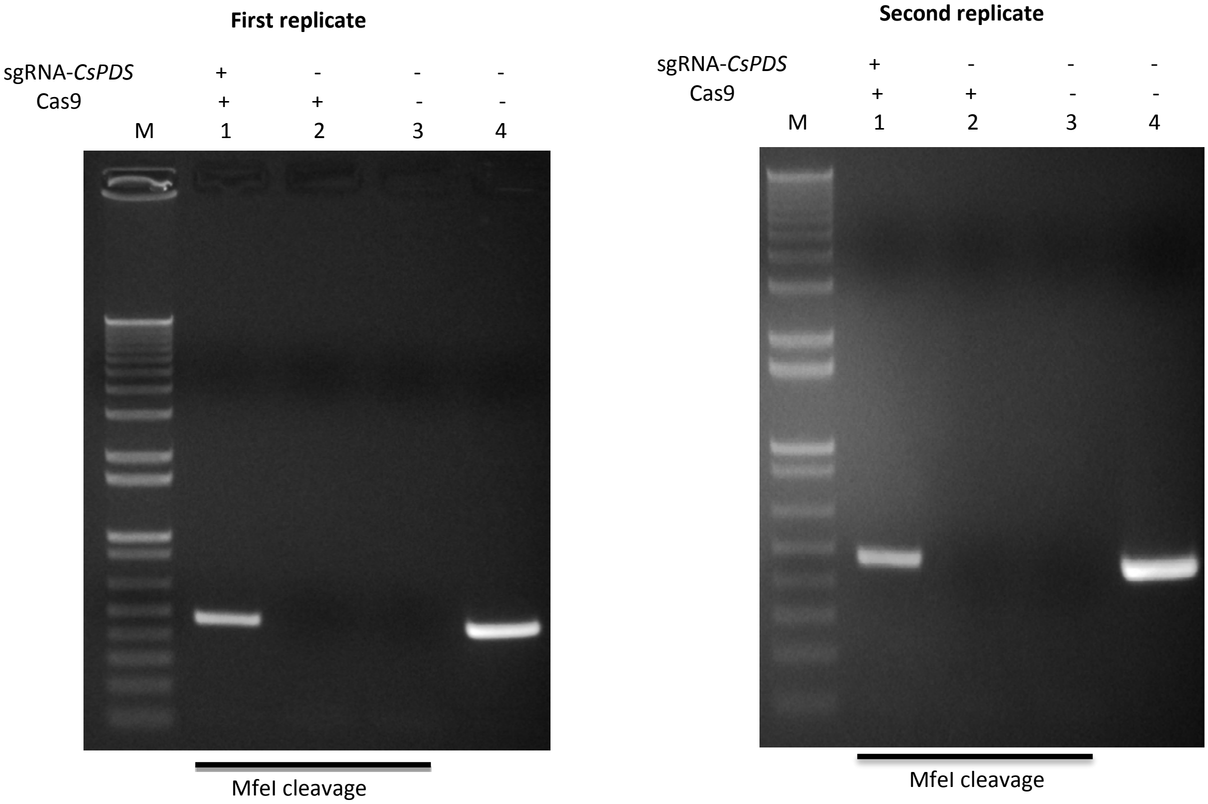

Supplementary Figure S2. Two replicates of the experiment presented in Figure 2 (b). Restriction-enzyme-digestion-suppressed PCR was used to detect the Cas9/sgRNA-induced mutation *in planta*. PCR amplification was conducted using the primers CsPDS-5-P1 and CsPDS-3-P2, which flank the target site within the *CsPDS* gene. Lanes 1-3, the template genomic DNA was digested with *Mfe*I. Lane 4, non-digested genomic DNA was used as a template. The PCR product in lane 1 resulted from Cas9/sgRNA-induced disruption of *Mfe*I, which indicates the expected disruption of the *Mfe*I site within the *CsPDS* gene. M, 1 kb DNA ladder.

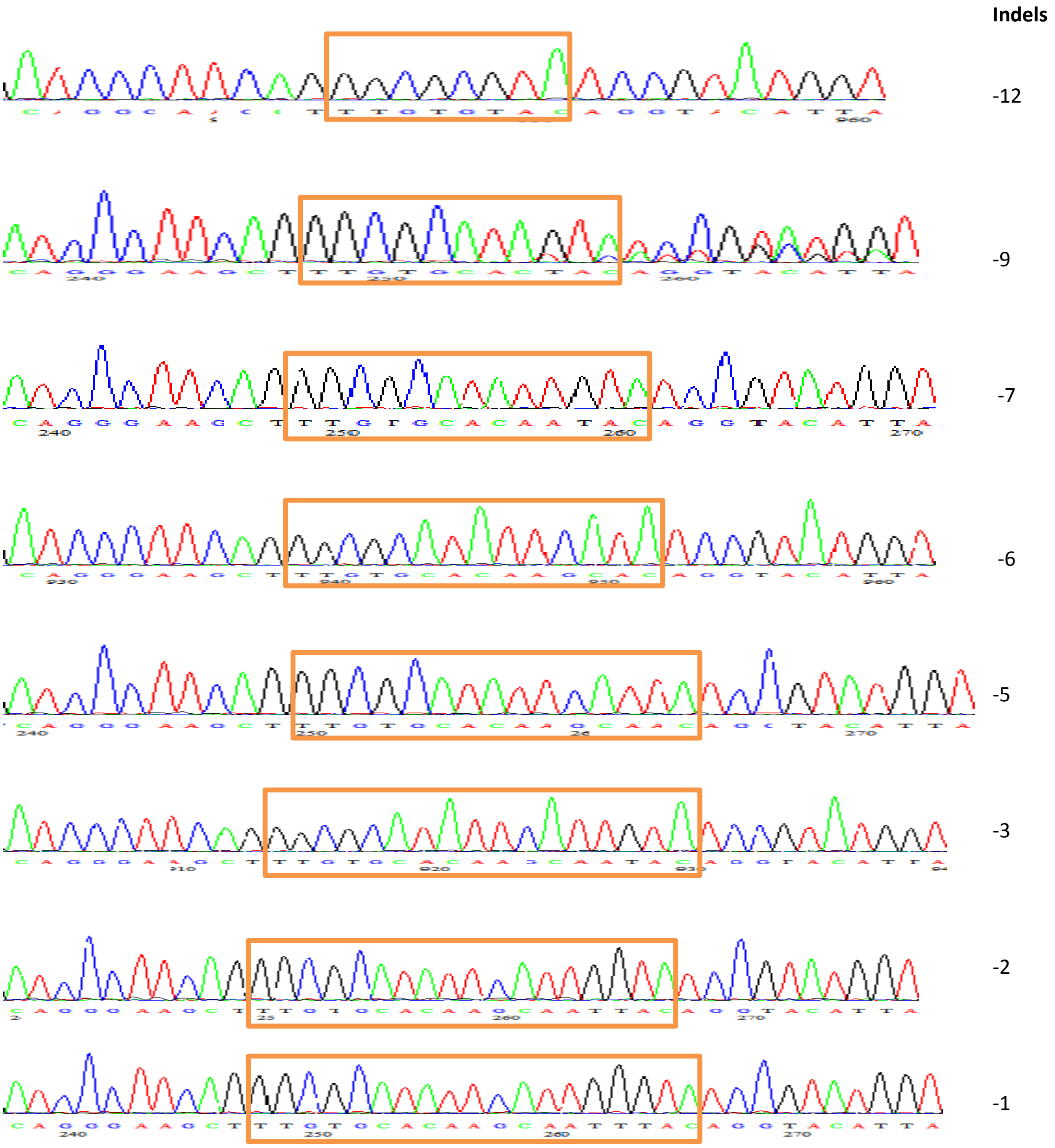

Supplementary Figure S3. The representative indel chromatograms of the *CsPDS12* mutations inducted by Cas9/sgRNA. The targete sequence within the *CsPDS* gene is highlighted by an orange rectangle.

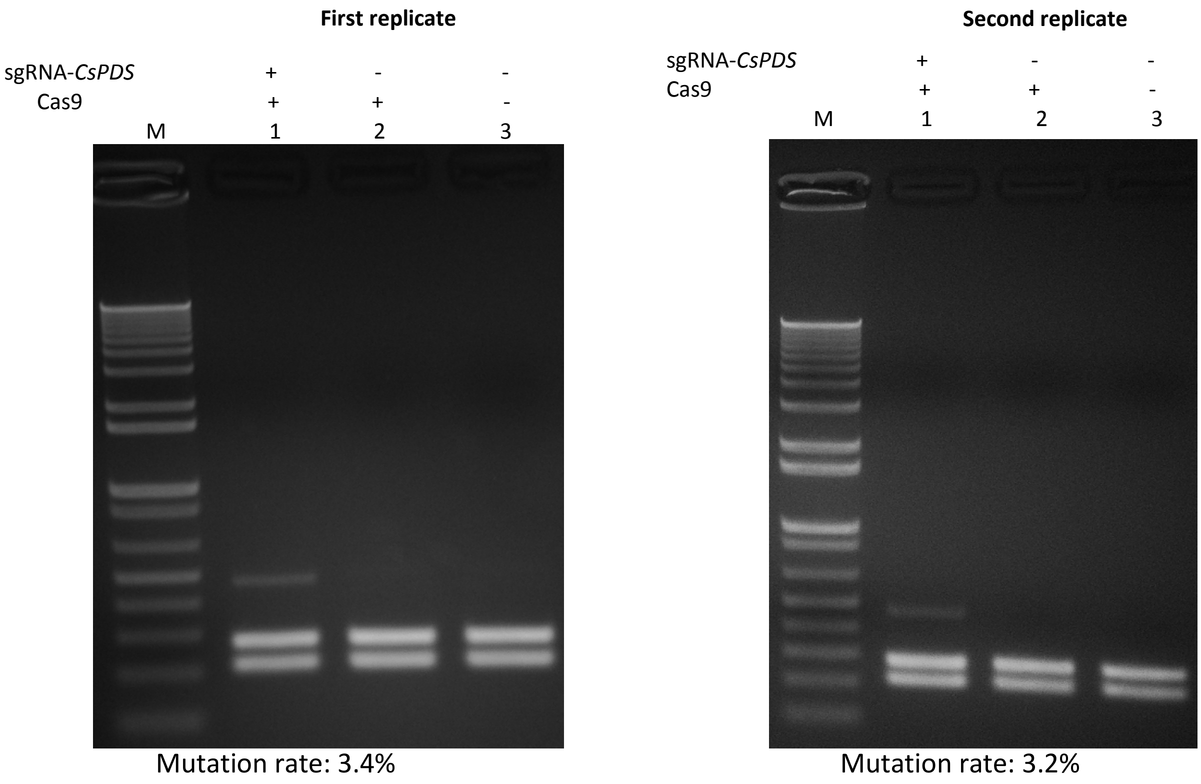

Supplementary Figure S4. Two replicates of the experiment presented in Figure 2 (d). Measurement of the mutation rate of the *CsPDS* gene induced by Cas9/sgRNA. After PCR amplification of the targeted PDS region, the products were subjected to *Mfe*I digestion. After separation on an agarose gel, the intensities of the bands were quantified using AlphaImager EP. The mutation rate was calculated by dividing the intensity of the uncut band by the intensity of all the bands in the lane. M, 1 kb DNA ladder.

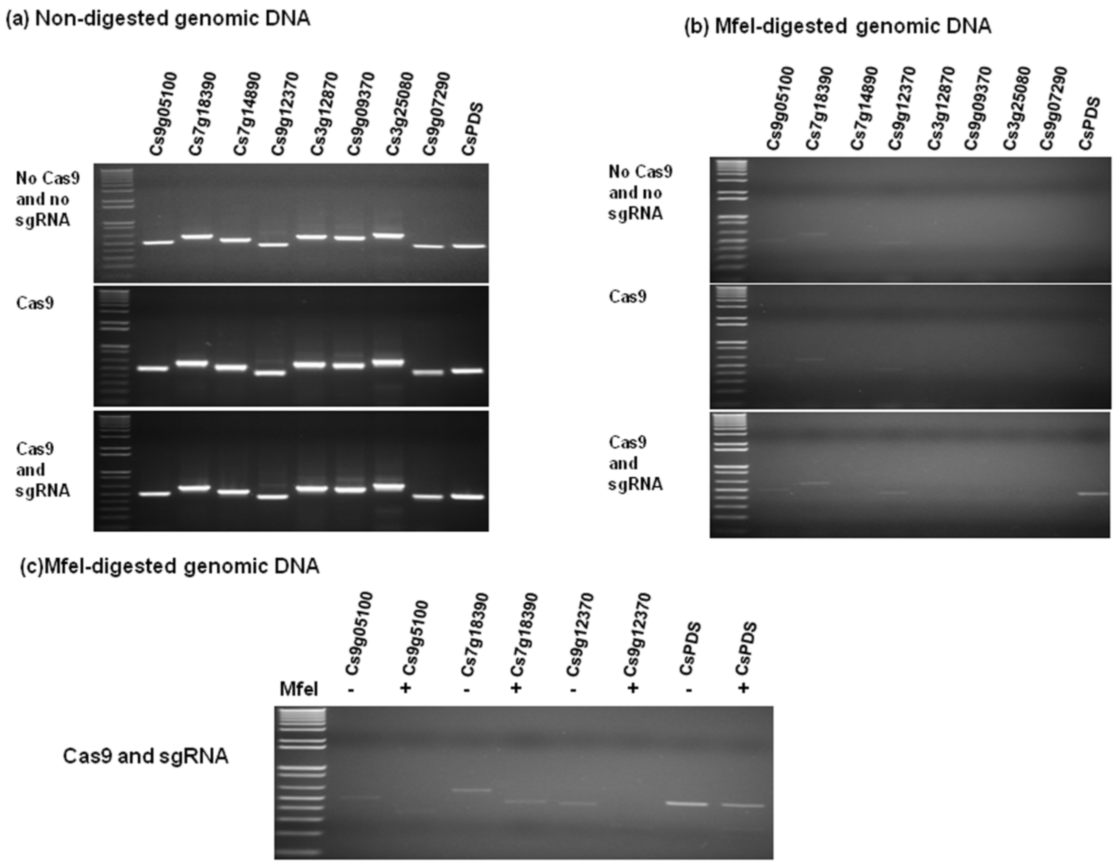

Supplementary Figure S5. Analysis of potential off-target sequences of the *CsPDS*-targeting Cas9/sgRNA by *Mfe*I-suppressed PCR. (a) Eight potential off-target sequences were amplified by PCR when non-digested genomic DNA was used as the template. (b) When *Mfe*I-digested genomic DNA was used, no PCR products or alleviated PCR products were observed. (c) After selective PCR amplification of mutagenized *CsPDS* genes, only the PCR product from the *CsPDS* gene showed resistance to *Mfe*I digestion. These results indicated that the *Mfe*I restriction sites in the 8 potential off-target sequences were not disrupted by Cas9/sgRNA cleavage and NHEJ repair.
